# A Unique Case of Lupus Erythematosus Profundus Presenting With Localized Scarring Alopecia and Perioral Swelling in a Pediatric Patient

**DOI:** 10.7759/cureus.105300

**Published:** 2026-03-16

**Authors:** Hasan Almoosawi, Fatima Samiey, Ameen Al Awadhi

**Affiliations:** 1 Dermatology, Salmaniya Medical Complex, Manama, BHR

**Keywords:** chronic cutaneous lupus erythematosus, dermatology case report, hydroxychloroquine, lupus erythematosus profundus, lupus panniculitis, pediatric case, pediatric lupus, scarring alopecia

## Abstract

Lupus erythematosus profundus, also known as lupus panniculitis, is a rare subtype of cutaneous lupus erythematosus characterized by inflammatory involvement of the deep dermis and subcutaneous adipose tissue. It is predominantly reported in adults, whereas pediatric presentations remain uncommon.

A five-year-old Middle Eastern girl presented with a two-month history of progressive localized alopecia involving the left parietal scalp, associated with perioral swelling predominantly affecting the upper lip. Examination showed an alopecic patch measuring approximately 4 × 2 cm with reduced follicular openings, suggestive of early scarring alopecia, and diffuse upper lip swelling with surrounding mild erythema. Trichoscopy demonstrated decreased follicular density and areas of absent follicles. Laboratory evaluation revealed elevated inflammatory markers with negative antinuclear antibody testing and normal complement levels. Histopathological examination of scalp and nasolabial fold biopsies demonstrated predominant lobular lymphocytic and histiocytic panniculitis extending into the subcutaneous fat, consistent with lupus erythematosus profundus. Treatment with oral hydroxychloroquine led to significant improvement in lip swelling and stabilization of the scalp lesion, with subsequent hair regrowth on follow-up.

Lupus erythematosus profundus is an uncommon but important cause of localized alopecia and facial swelling in children. Early clinicopathologic recognition and timely treatment are essential to reduce the risk of permanent scarring and disfigurement.

## Introduction

Lupus erythematosus profundus, also referred to as lupus panniculitis, is an uncommon variant of cutaneous lupus erythematosus defined by chronic inflammatory involvement of the deep dermis and subcutaneous adipose tissue, most commonly presenting as predominantly lobular panniculitis [1]. Clinically, it typically presents as deep-seated indurated nodules or plaques that may resolve with lipoatrophy, scarring, and cosmetic deformity. Lupus erythematosus profundus may occur either as an isolated cutaneous condition or in association with discoid lupus erythematosus and systemic lupus erythematosus [1-3]. Its pathogenesis remains not fully understood, but immune-mediated tissue injury characterized by lymphocytic inflammation and immune complex deposition within the subcutaneous tissue is considered central to disease development [1,4].

The prevalence of cutaneous lupus erythematosus has been estimated at approximately 70 cases per 100,000 persons, while lupus erythematosus profundus represents a relatively small proportion of these cases [2,4]. Pediatric disease is particularly rare. In a review published in 2012, only 12 well-documented pediatric cases had been identified in the literature in the English language prior to the authors reporting three additional pediatric cases [5]. Subsequent pediatric reports have remained infrequent, including childhood cases associated with discoid lupus erythematosus, linear lupus erythematosus profundus as an initial presentation of systemic lupus erythematosus, and scalp-predominant linear or annular variants [6-8].

The condition most commonly involves the face, proximal extremities, trunk, buttocks, and breasts [1,4]. However, scalp involvement is also recognized and may present a diagnostic challenge, particularly when alopecia is the predominant clinical feature [7,8]. Because its clinical morphology overlaps with several inflammatory, granulomatous, and infiltrative disorders, histopathological examination is essential for establishing the diagnosis. We report a rare pediatric case of lupus erythematosus profundus presenting with localized scarring alopecia and perioral swelling, an unusual combination, which expands the clinical spectrum of pediatric lupus profundus and highlights the importance of early biopsy in atypical childhood presentations.

## Case presentation

A five-year-old Middle Eastern girl presented to the dermatology department at Salmaniya Medical Complex with a two-month history of progressive localized hair loss over the left parietal scalp. According to the history, the lesion had begun as a smaller focal patch and gradually enlarged over the preceding two months. There was no reported prior episode of similar alopecia, no documented recurrence before this presentation, and no associated pruritus, pain, discharge, trauma, or preceding infection. The patient had also developed progressive perioral swelling, predominantly involving the upper lip, with mild surrounding erythema. There was no history of photosensitivity, oral ulcers, arthralgia, fever, weight loss, Raynaud's phenomenon, seizures, hematuria, edema, or other symptoms suggestive of systemic lupus erythematosus. Past medical, surgical, and drug histories were unremarkable. Family history was non-contributory.

**Figure 1:**
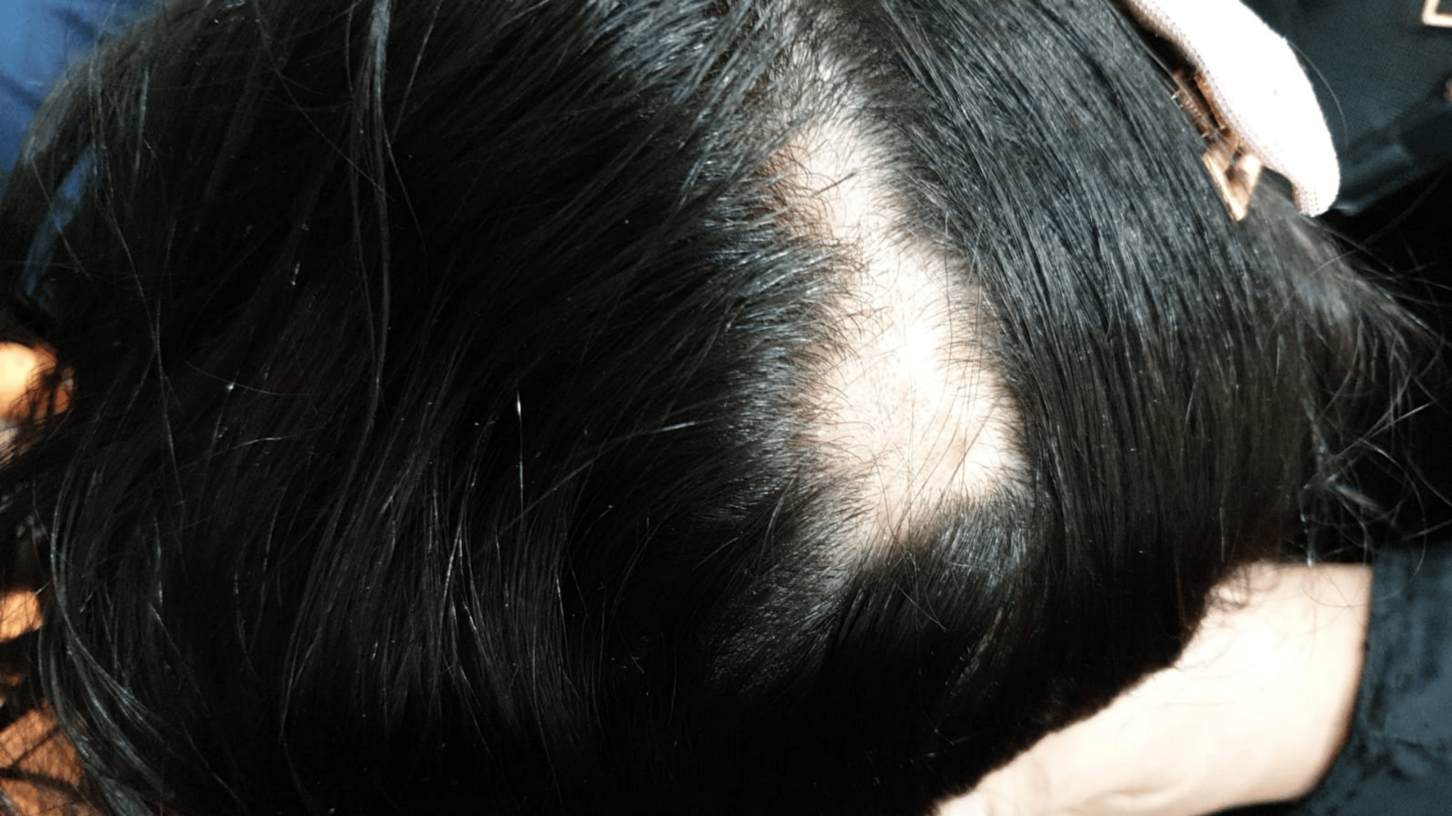
Localized alopecic patch over the left parietal scalp measuring approximately 4 × 2 cm, with reduced follicular openings suggestive of early scarring alopecia

On general examination, the patient appeared well, was vitally stable, and had age-appropriate growth parameters. Cutaneous examination demonstrated a localized alopecic patch over the left parietal scalp measuring approximately 4 × 2 cm. The surface was smooth with reduced follicular openings and without appreciable scale, crust, pustulation, ulceration, or discharge. The lesion appeared clinically indurated rather than fluctuant and was most consistent with an early inflammatory cicatricial process. Figure 1 shows the scalp lesion. Trichoscopy demonstrated decreased follicular density and areas of absent follicles, in keeping with early scarring alopecia.

**Figure 2:**
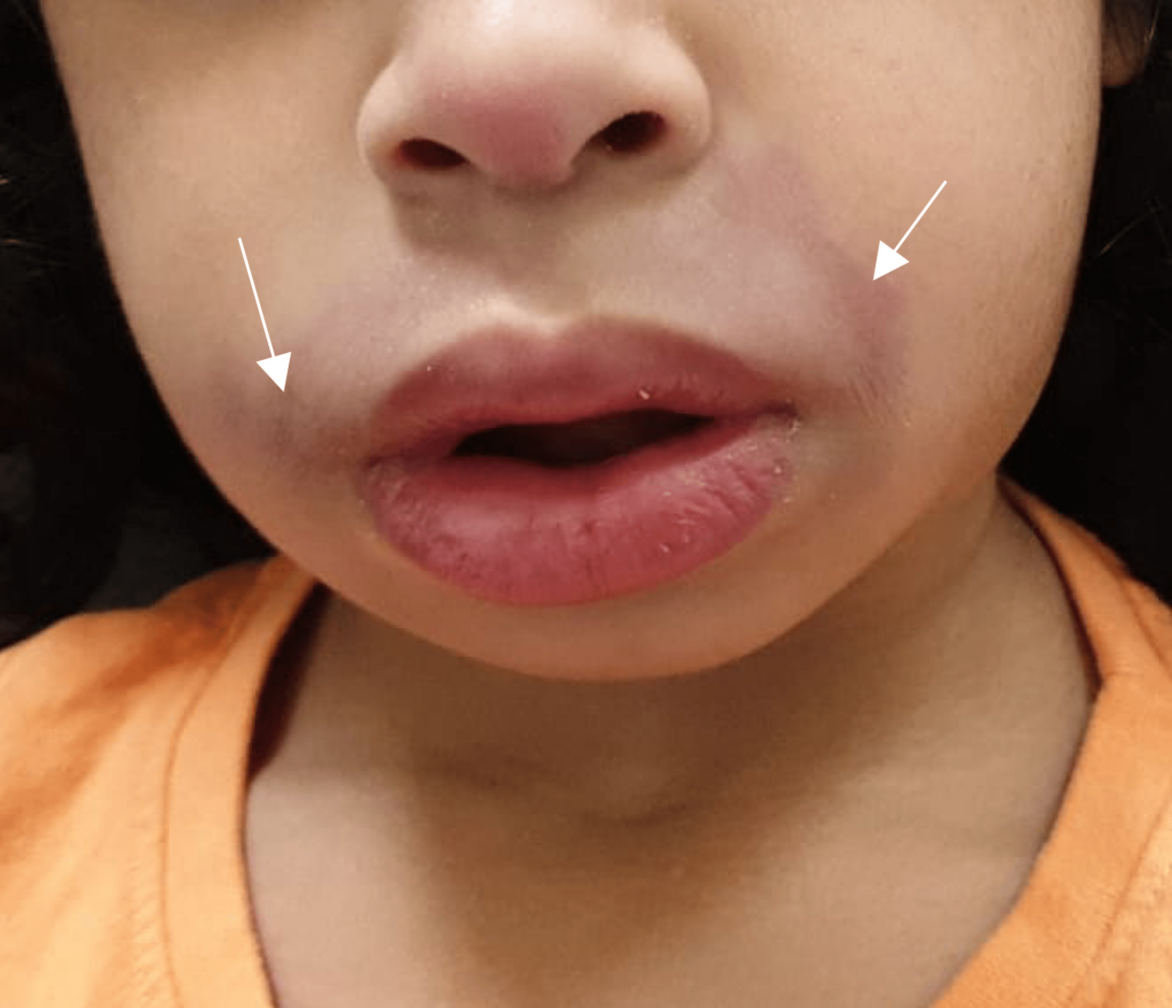
Diffuse swelling of the upper lip with surrounding mild erythematous change

Examination of the perioral region showed diffuse swelling of the upper lip with mild surrounding erythematous patches. Figure 2 shows the facial involvement. Palpation of the neck revealed small bilateral cervical lymph nodes, measuring approximately 1 cm in diameter, that were mobile and non-tender.

Laboratory investigations demonstrated elevated erythrocyte sedimentation rate and C-reactive protein levels. Antinuclear antibody testing was negative; anti-double-stranded DNA antibodies were negative, and complement levels, including C3 and C4, were within normal limits. Complete blood count, liver function tests, renal function tests, and urinalysis were all within normal ranges. Chest radiography did not reveal any abnormalities.

**Figure 3:**
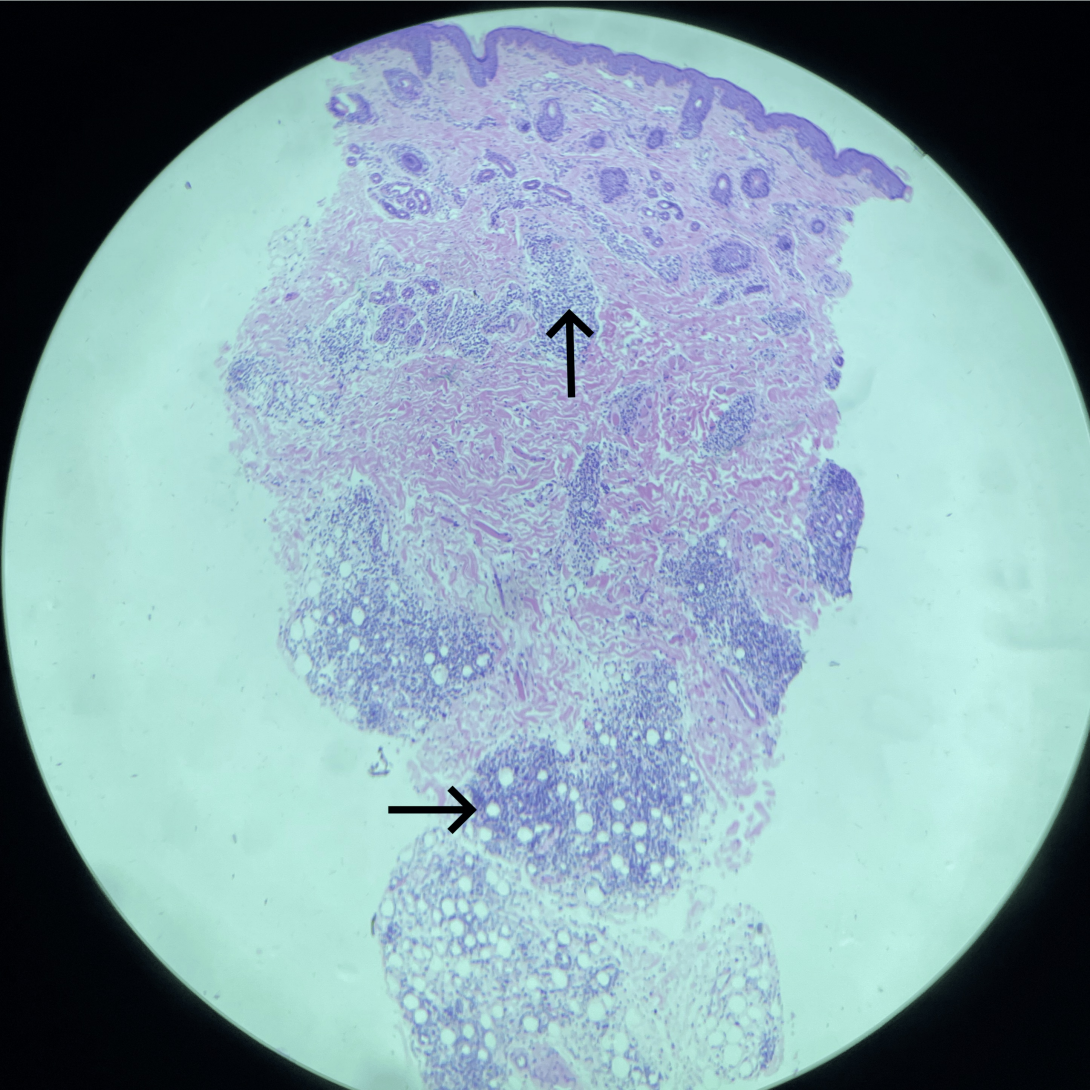
Skin punch biopsy from the face measuring 0.4 cm in diameter and 0.4 cm in length Low-power histopathological image demonstrating unremarkable epidermis. Upper and lower dermis show perivascular lymphocytic and histiocytic infiltrates extending to the subcutaneous tissue, involving mainly lobules. Unremarkable epidermis. Upper and lower dermis show perivascular lymphocytic and histiocytic infiltrates. Same infiltrates as above are seen extending to the subcutaneous tissue, involving mainly lobules

**Figure 4:**
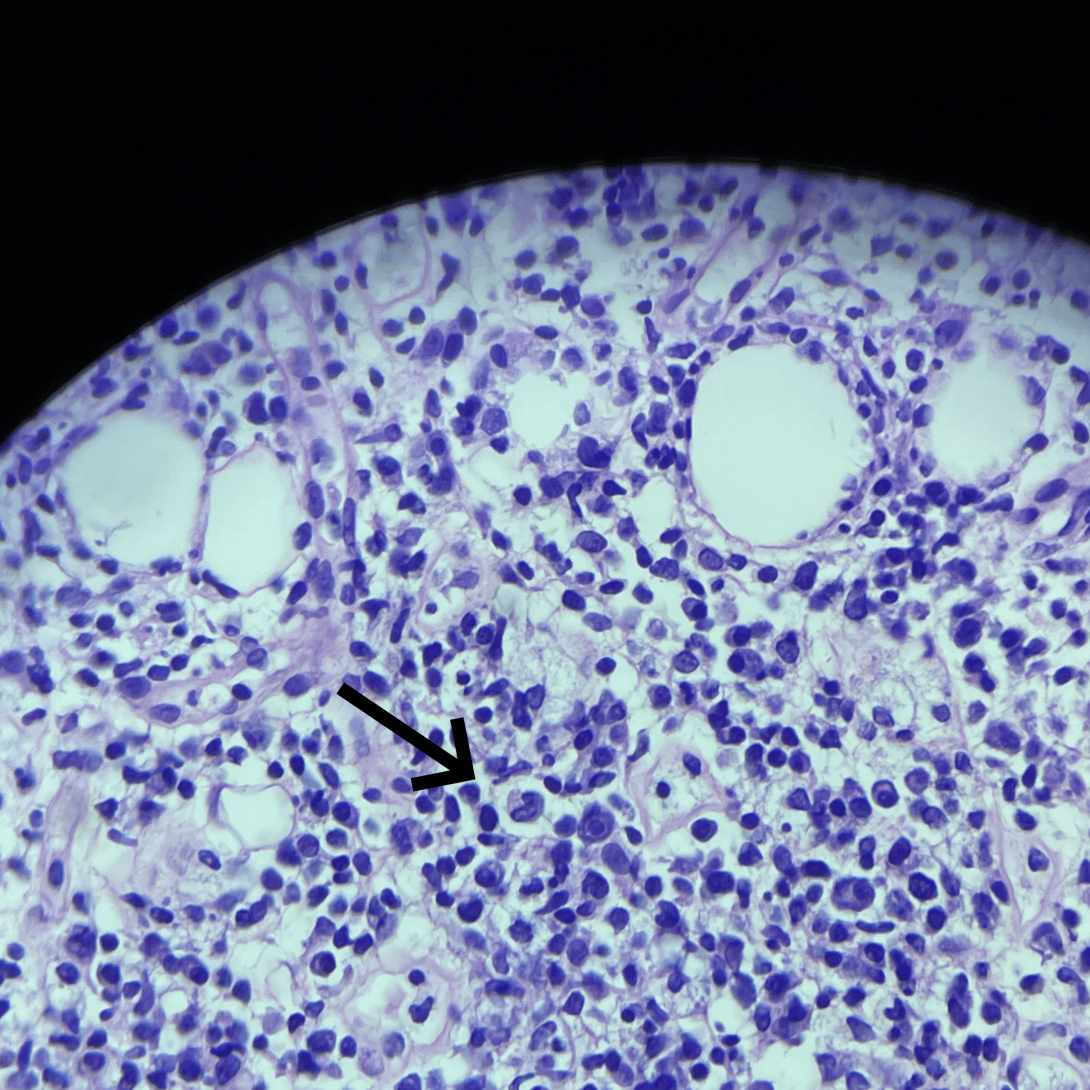
Skin punch biopsy from the scalp measuring 0.5 cm in diameter and 0.4 cm in length Higher-power histopathological image showing predominantly lobular lymphocytic and histiocytic infiltrates within the subcutaneous fat

Two punch biopsies measuring 4 mm each were obtained under sedation from the scalp and nasolabial lesions. Histopathological examination showed an unremarkable epidermis. The upper and lower dermis demonstrated perivascular lymphocytic and histiocytic inflammatory infiltrate extending into the subcutaneous tissue. The panniculitic component was predominantly lobular in distribution, supporting the diagnosis of lupus erythematosus profundus. No definite alternative granulomatous process was identified. If specifically noted in the pathology report, the presence or absence of hyaline fat necrosis or mucin deposition may be stated here. Figures 3-4 demonstrate representative histopathological findings.

Immunohistochemical staining demonstrated CD3- and CD20-positive lymphoid cells in a reactive distribution, while kappa and lambda stains supported a polyclonal pattern. CD30 highlighted scattered immunoblasts only, without any evidence of a lymphoproliferative process. Based on the clinical presentation, trichoscopic findings, and histopathological features, a diagnosis of lupus erythematosus profundus involving the scalp and perioral region was made.

**Figure 5:**
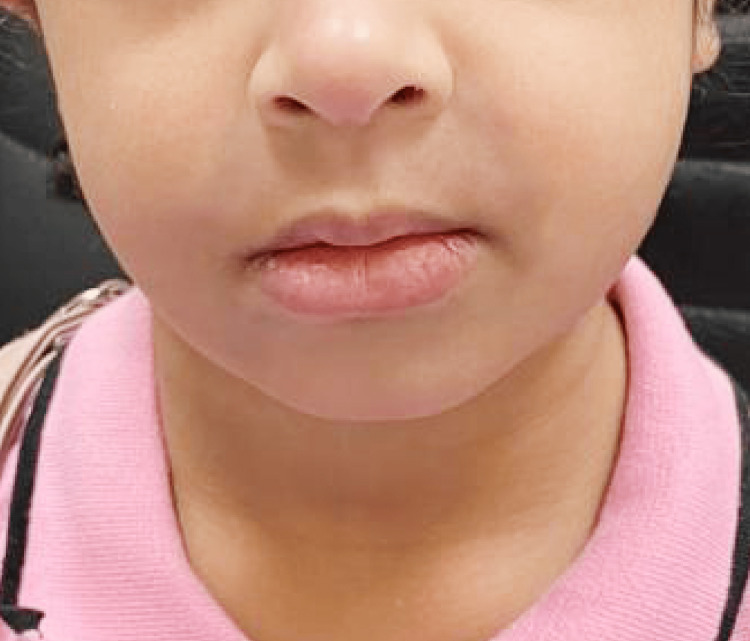
Clinical improvement in upper-lip swelling after the initiation of hydroxychloroquine therapy

**Figure 6:**
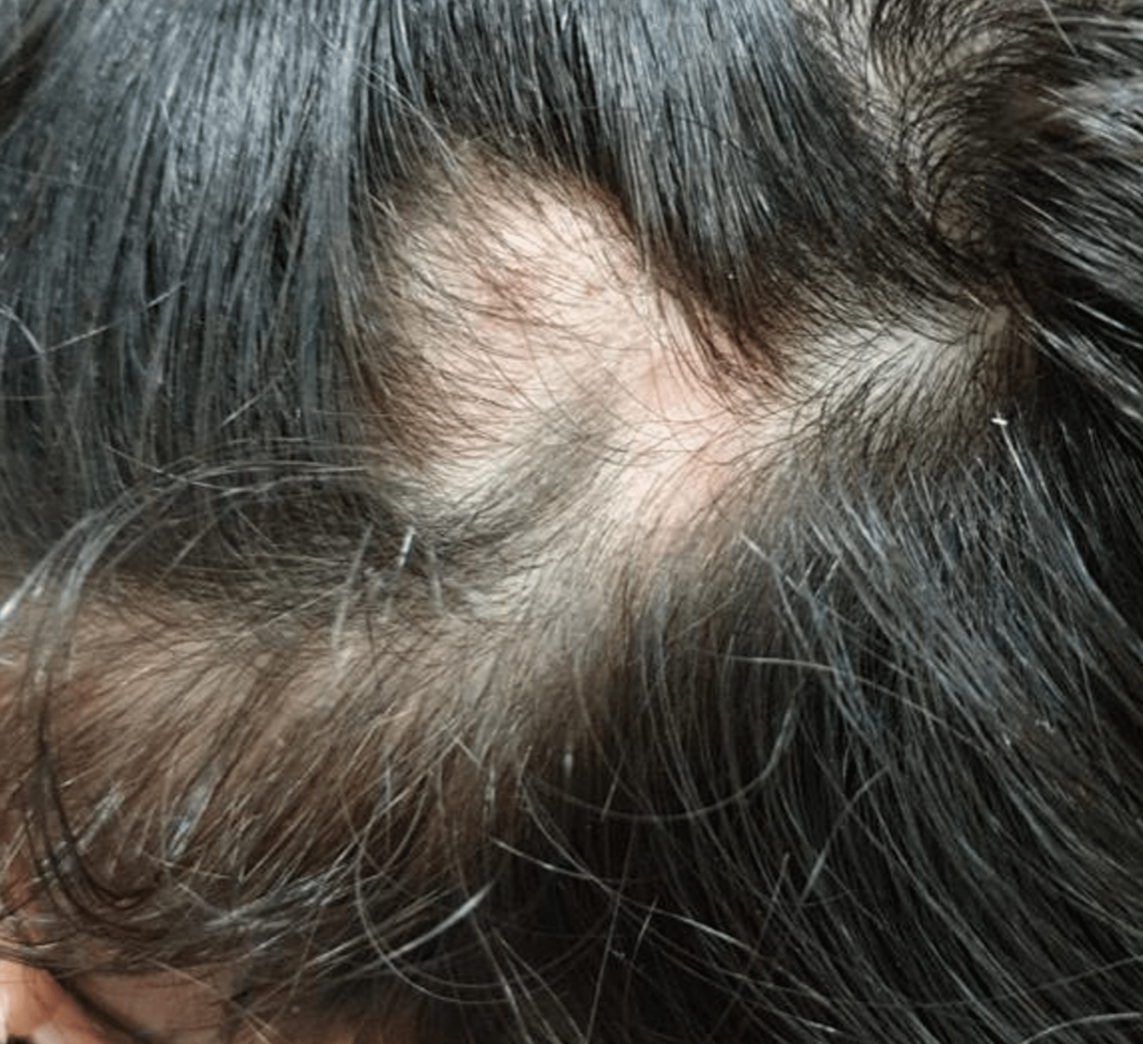
Follow-up scalp photograph demonstrating interval hair regrowth and stabilization of the alopecic patch

The patient was started on oral hydroxychloroquine 100 mg once daily. On follow-up, there was marked improvement in upper-lip swelling within the early treatment period, with subsequent stabilization of the scalp lesion. Follow-up photographs showed interval hair regrowth in the involved scalp area. Figures 5-6 demonstrate the clinical response.

## Discussion

Lupus erythematosus profundus represents a rare but distinctive form of cutaneous lupus erythematosus in which inflammation preferentially targets the deep dermis and subcutaneous fat [1]. Histopathologically, the process is typically characterized as a predominantly lobular lymphocytic panniculitis, often accompanied by variable dermal and periappendageal inflammation, hyaline fat necrosis, mucin deposition, and lymphoid aggregates [1,4]. In clinical practice, the diagnosis may be difficult when some classic histologic features are absent or only partially represented, which makes clinicopathologic correlation especially important.

Our patient’s presentation was unusual for several reasons. First, pediatric lupus erythematosus profundus is distinctly uncommon. A 2012 pediatric review identified only 12 previously reported pediatric cases in the English literature before adding three additional cases [5]. Second, the combination of localized scalp alopecia and conspicuous perioral swelling is atypical. Third, the scalp lesion showed features of early scarring alopecia, raising an immediate differential diagnosis that extended beyond lupus to include inflammatory cicatricial alopecias and infiltrative processes. The clinical differential diagnosis in this child appropriately included sarcoidosis, orofacial granulomatosis, cutaneous lupus erythematosus, mucinosis, amyloidosis, and hyalinosis cutis et mucosae. The absence of systemic symptoms, the lack of an alternative granulomatous pattern on biopsy, and the demonstration of a predominantly lobular lymphocytic panniculitis with supportive immunohistochemistry favored a diagnosis of lupus erythematosus profundus.

The literature also demonstrates the heterogeneity of pediatric lupus profundus. Wimmershoff et al. described childhood lupus profundus associated with discoid lupus erythematosus [6]. Fernandes et al. reported linear lupus erythematosus profundus in a child as the initial manifestation of systemic lupus erythematosus, highlighting that systemic progression, although uncommon, may occur [7]. Scalp-predominant pediatric variants have also been described, including linear and annular forms presenting with alopecia, which may mimic other scalp disorders and thereby delay diagnosis [7,8]. Compared with those reports, our case is notable for its combined presentation of localized alopecia and perioral swelling and for the favorable response to hydroxychloroquine monotherapy.

Serological abnormalities in lupus erythematosus profundus can be variable. A positive antinuclear antibody test may be seen in a substantial proportion of patients with cutaneous lupus erythematosus, but a negative antinuclear antibody result does not exclude lupus profundus, especially when the disease is confined to the skin [2,3]. In our patient, the lack of constitutional symptoms, the absence of mucosal or joint manifestations, a negative antinuclear antibody, and normal complement levels favored the presence of isolated cutaneous disease at presentation rather than overt systemic lupus erythematosus.

Elevated erythrocyte sedimentation rate and C-reactive protein in this context are nonspecific inflammatory markers. They indicate the presence of active inflammation but cannot alone confirm systemic disease. The reviewer’s comment regarding common anatomic sites is important. Classic sites for lupus erythematosus profundus include the face, proximal extremities, trunk, breasts, and buttocks [1,4]. Although the scalp is not usually emphasized as the most common site overall, scalp involvement is clearly recognized and is particularly relevant when lupus profundus presents with alopecia. Later reports have indeed highlighted scalp-predominant linear and annular variants as a distinct clinical pattern [8].

Treatment recommendations are based largely on case series and expert reviews rather than large trials because of the rarity of the disease. Antimalarials, particularly hydroxychloroquine, are widely regarded as first-line systemic therapy for cutaneous lupus erythematosus and lupus profundus due to their immunomodulatory effects and generally favorable safety profile [2,9]. Systemic corticosteroids may be used in more inflammatory or rapidly progressive disease, while methotrexate, azathioprine, mycophenolate mofetil, dapsone, or other steroid-sparing agents may be considered in refractory cases [2,10]. In our case, hydroxychloroquine alone produced clear clinical benefit, with marked improvement in lip swelling and subsequent hair regrowth, supporting its use as initial therapy.

The long-term risk of progression from isolated lupus erythematosus profundus to systemic lupus erythematosus remains unclear but is generally low; however, progression has been reported, including in pediatric patients [5,7]. For this reason, continued longitudinal follow-up is advisable, with reassessment for systemic symptoms and repeat laboratory testing when clinically indicated.

The uniqueness of this case

This case is unusual because lupus erythematosus profundus remains rare in childhood and because the presenting combination of localized scalp alopecia and perioral swelling does not represent a classic phenotype. The case also demonstrates that an early biopsy can establish the diagnosis before more extensive scarring develops. Finally, the favorable response to hydroxychloroquine highlights the importance of prompt recognition and treatment to minimize permanent alopecia and facial disfigurement.

## Conclusions

This report describes a rare pediatric presentation of lupus erythematosus profundus, manifesting as localized inflammatory scalp alopecia and perioral swelling. It emphasizes that lupus profundus should be considered in children with atypical alopecia accompanied by facial soft-tissue swelling, even when antinuclear antibody is negative, and other overt features of systemic lupus erythematosus are absent. Early histopathological confirmation and timely initiation of treatment, particularly with hydroxychloroquine, may stabilize disease activity, improve cosmetic outcomes, and reduce the likelihood of permanent scarring. Ongoing follow-up remains important because future systemic evolution, although uncommon, cannot be entirely excluded.
